# Acquisition of novel antibiotic resistance genes by the bacterial predator *Bacteriovorax* sp. As-1

**DOI:** 10.1093/ismejo/wraf245

**Published:** 2025-11-03

**Authors:** Fathrinah Binti Kohadie, Young-Ung Heo, Wonsik Mun, Sumin Choi, Sinseong Park, Yoonhang Lee, Do-Hyung Kim, Robert J Mitchell

**Affiliations:** Department of Biological Sciences, Ulsan National Institute of Science and Technology (UNIST), Ulsan 44919, Republic of Korea; Department of Aquatic Life Medicine, Pukyong National University, Busan 48513, Republic of Korea; Department of Biological Sciences, Ulsan National Institute of Science and Technology (UNIST), Ulsan 44919, Republic of Korea; Department of Biological Sciences, Ulsan National Institute of Science and Technology (UNIST), Ulsan 44919, Republic of Korea; Department of Biological Sciences, Ulsan National Institute of Science and Technology (UNIST), Ulsan 44919, Republic of Korea; Department of Aquatic Life Medicine, Chonnam National University, Yeosu 59626, Republic of Korea; Department of Aquatic Life Medicine, Pukyong National University, Busan 48513, Republic of Korea; Department of Biological Sciences, Ulsan National Institute of Science and Technology (UNIST), Ulsan 44919, Republic of Korea

**Keywords:** *Bacteriovorax*, bacterial Predation, horizontal gene transfer, antibiotic resistance

## Abstract

This study reports the isolation and characterization of *Bacteriovorax* sp. As-1, a predatory bacterium recovered from the gut of oxytetracycline-treated juvenile rainbow trout (*Oncorhynchus mykiss*). Phylogenetic and genomic analysis indicate it is closely related to *Bacteriovorax stolpii* DSM 12778^T^, although genomic metrics suggest it represents a new species. Like other *Bdellovibrio*-and-like organisms, *Bacteriovorax* sp. As-1 exhibits predatory activity against *Aeromonas salmonicida*, significantly reducing its prey viability by nearly six orders of magnitude. However, whole genome sequencing revealed the presence of multiple antibiotic resistance genes, including those previously associated with decreased susceptibility to tetracyclines, aminoglycosides, sulfonamides, and fluoroquinolones, located within genomic islands, and flanked by insertion sequences, suggesting acquisition via horizontal gene transfer (HGT). In addition to these, mutations were also detected in *gyrA* gene that confer resistance to ciprofloxacin. Phenotypic assays confirmed *Bacteriovorax* sp. As-1 has increased antibiotic resistance as compared to *Bx. stolpii* DSM 12778^T^. This study presents a natural predatory strain carrying IS-linked ARG clusters consistent with HGT, highlighting their potential role as reservoirs of resistance determinants in antibiotic-enriched environments.

## Introduction

The overuse of antibiotics poses a significant threat to environmental and organismal health [1] and ecological processes [2, 3]. Within aquaculture, antibiotics are often administered prophylactically to prevent disease outbreaks [4–6]. However, due to their low bioavailability and limited absorption and uptake, a substantial fraction is excreted into the surrounding environment [7, 8], where it drives the emergence and spread of antibiotic-resistant bacteria [9]. The problem is further exacerbated by the extensive and sometimes indiscriminate use of antibiotics. Between 2008 and 2018, the top 15 aquaculture-producing countries collectively used 67 different antibiotics, including those banned in certain regions (penicillin, chloramphenicol, and rifamycin in China, Vietnam, and Thailand) [10]. Three of the approved antibiotics (i.e. oxytetracycline, florfenicol, or sulphadiazine) were used by more than half of these countries [10], including South Korea [11].

The consequences of such antibiotic use on microbial communities are well documented. For instance, the oral administration of florfenicol significantly increased the relative abundance of antibiotic resistance genes (ARGs) and mobile genetic elements (MGEs) in the gut microbiota of pacu (*Piaractus mesopotamicus*) [12]. Similarly, the dietary supplementation with oxytetracycline (OTC) or sulfadiazine/trimethoprim (SDZ/TMP) in juvenile rainbow trout (*Oncorhynchus mykiss*) increased the relative abundance of *tet* and *sul*/*dfr* genes, respectively in the gut microbiome [13]. These findings highlight the potential risks associated with antibiotic use in aquaculture, particularly its role in facilitating ARG dissemination. Among the microbial populations affected by OTC exposure, predatory bacteria were ranked among the top 20 bacterial taxa present in the gut of rainbow trout [13].

Predatory bacteria, including members of the genera *Bdellovibrio* [14, 15] and *Bacteriovorax* [16], are distinguished by their ability to prey upon a broad range of Gram-negative bacteria [17]. Collectively referred to as *Bdellovibrio*-and-like organisms (BALOs), these bacteria are naturally abundant in diverse ecosystems, including soil, marine, and freshwater environments, where they play a pivotal role in shaping microbial communities [18–21]. Studies indicate BALOs thrive in environments with high bacterial diversity and prey density, often targeting pathogenic bacteria [21–23]. Towards this end, several predators have been successfully used as biocontrol agents in the aquaculture industry, reducing the incidence and severity of infectious diseases in fish and shellfish [14, 24]. Their potential to control bacterial pathogens without the use of traditional antibiotics has been viewed as a promising strategy to enhance economic sustainability and food safety in aquaculture [17, 24, 25].

BALOs are sometimes referred to as “living antibiotics” in the literature due to these benefits [26–28]. However, unlike conventional antibiotics, BALOs are biological entities that require suitable environmental conditions and prey availability to function effectively. Despite their antimicrobial potential, BALOs are themselves susceptible to conventional antibiotics, particularly translation inhibitors, such as tetracyclines [29, 30]. This includes OTC [31], making the recent findings with rainbow trout particularly intriguing; after OTC treatment, the relative abundance of predatory bacteria in the rainbow trout gut increased nearly 200% [13]. Although this surge could be partially explained by the reestablishment of the gut microbiome, leading to an enrichment of specific prey species such as *Flavobacterium* and *Pseudomonas* [13, 23], it does not fully account for the sustained presence of BALOs during antibiotic treatment. One plausible explanation is that OTC exposure not only enriched ARG pools but also increased horizontal gene transfer (HGT) rates in the gut microbiome [13]. This raises a critical question: can BALOs, when exposed to antibiotic-enriched environments, acquire ARGs and thereby gain a survival advantage? Even though evidence exists for ARG transfer to other predatory bacteria (*Myxococcota* [32]), direct evidence of recent ARG acquisition by natural BALO isolates remains limited. This study therefore investigates whether BALOs can serve as recipients of ARGs, evaluates their association with reduced susceptibility, and considers the potential ecological risks posed by antibiotic-resistant predatory bacteria.

## Materials and methods

### Fecal sampling

Juvenile rainbow trout (*O. mykiss*) were fed commercial dry pellets supplemented with oxytetracycline (OTC; 75 mg/kg of body weight) as reported earlier [13]. Fecal samples were collected from each tank using the siphoning method during the water exchange process, following a previously described protocol [13]. To ensure consistency, 100% of the water was replaced daily, 24 h after fish feeding, throughout the experimental period. At each sampling time point, fecal samples were retrieved and immediately processed to remove residual water. The collected samples were centrifuged at 8000 rpm for 3 min at 15°C, supplemented with 10% (v/v) glycerol, and stored at −80°C for further analyses.

### Prey culture preparation

*Aeromonas salmonicida* subsp. *salmonicida* BB21151NE was isolated in 2021 from the skin of a rainbow trout collected at a trout farm in Chuncheon, Korea. Genomic DNA was extracted from the purified strain using the AccuPrep Genomic DNA Extraction Kit (Bioneer, Korea). The bacterial identity was confirmed via PCR amplification of the 16S rRNA gene using a universal primer set (27F: 5′-GAGTTTGATCCTGGCTCAG-3′, 1492R: 5′-GGTTACCTTGTTACGACTT-3′). Subspecies classification was further verified with a *vapA*-specific primer set (A-layer 2: 5′- TCTGCGTCTTGCTCTTGCT -3′, R3: 5′- ACGTTGGTAATCGCGAAATC -3′) [33]. This fish pathogen served as the prey bacterium for predatory bacterial isolation and was utilized in various assays throughout the study. The prey bacteria were cultivated in tryptic soy broth (TSB) at 25°C with constant shaking at 250 rpm overnight. Bacterial cells were then harvested by centrifugation (4200 × g, 10 min), washed with sterilized HEPES buffer (25 mM, pH 7.2) supplemented with 3 mM MgCl_2_ and 2 mM CaCl_2_, and resuspended to an initial optical density (OD_600_) of 1.0.

### Isolation and identification of *Bacteriovorax* sp. As-1

The fecal samples from the rainbow trout were thawed on ice and diluted (100-fold) into sterile HEPES buffer (25 mM, pH 7.2 with 3 mM MgCl_2_ and 2 mM CaCl_2_) and added (1:100 dilution) to the prepared prey culture. This culture was incubated at 30°C with shaking at 250 rpm and the culture turbidities were observed daily until the OD dropped to a value below 0.3, indicating predation had occurred. Samples from these tubes were serially diluted in sterile HEPES buffer and added to 10 ml molten (45°C) top agar (0.7% agar prepared in sterile HEPES) with the prey at an initial OD of 1.0 as described previously [15]. The plates were then incubated at 30°C for up to three days, during which plaques formed. A single plaque with clearly defined edges was transferred into a fresh prey suspension in HEPES buffer prepared as above with an initial OD of 1.0. This culture was incubated at 30°C and 250 rpm until the OD dropped to a value below 0.3, which typically took one to 2 days. The culture was then filtered (0.45 μm polyether sulfone (PES); Millipore) to remove any remaining *A. salmonicida* or bdelloplasts, and the filtrate, which contained the predator, was mixed (1:1 (v:v)) with freshly grown *A. salmonicida* in HEPES buffer. After a 2-h incubation to allow bdelloplasts to form, 25% glycerol stocks were prepared, and these were stored at −80°C. As needed, fresh plaques of the predatory isolate were grown as described above using *A. salmonicida* as a prey. The purity and identity of this newly isolated predator was determined through PCR using the 27F/1492R primer set, which identified this isolate as a strain of *Bacteriovorax*. This isolate was designated as *Bacteriovorax* sp. As-1. All predators used in this study were grown using the basic protocols as described previously [15, 22].

### Whole genome sequencing and genomic analysis

Genomic DNA from *A. salmonicida* subsp. *salmonicida* BB21151NE (prey) and *Bacteriovorax* sp. As-1 (predator) was extracted using different procedures. Prey DNA was isolated with the Qiagen DNeasy 96 PowerSoil Pro QIAcube HT Kit (Qiagen, Germany); cells were transferred to MagMAX Microbiome Bead Beating Tubes (Thermo Fisher Scientific, USA) and mixed with CD1 Lysis Buffer prior to bead-beating (5 min, optimized for hybrid sequencing). Predator DNA was extracted with the Genomic DNA Isolation, Flexible Kit (Nucleogen, South Korea) following the manufacturer’s instructions.

For prey sequencing, a hybrid approach was employed: libraries were prepared with the DNA Prep tagmentation kit (Illumina, USA) and sequenced on the NextSeq 2000 System (Illumina) with a 300-cycle XLEAP-SBS flow cell to generate 2 × 150 bp paired-end reads, with 1%–2% PhiX spike-in as an internal control. Long-read libraries were prepared with the ONT Native Barcoding Kit 24 (#SQK-NBD114.24) and sequenced on the PromethION 2 Solo platform with FLO-PRO114M (R10.4.1) flow cells. Predator sequencing was performed exclusively with long reads, using the same barcoding kit, and run on a MinION device equipped with FLO-MINI114 (R10.4.1) flow cells.

Sequencing was conducted with MinKNOW v24.11.8, and base calling was performed with Dorado v7.6.7 in super-accurate mode with barcode trimming enabled. Read quality was inspected with NanoPlot v1.43.0 [34], and low-quality reads were filtered with Filtlong v0.2.1 (https://github.com/rrwick/Filtlong). Hybrid assemblies of the prey genome were generated with Flye v2.9.5 (Nanopore reads) [35], polished with Medaka v2.0.0 (https://github.com/nanoporetech/medaka), and further corrected with Illumina short reads. The predator genome was assembled de novo with Flye and polished with Medaka.

Assembly quality and completeness were assessed using QUAST v5.2.0, CheckM, and BUSCO v5.7.1 [36, 37], with potential contamination further evaluated by mapping Illumina short reads against prey-derived reads. Genome annotation for both strains was performed with Prokka (Galaxy ver. 1.14.6 + galaxy1) [38] and the Rapid Annotation using Subsystem Technology (RAST) server [39], and the final annotated assemblies were visualized with DNAPlotter from Artemis version 18.2.0 [40]. Genome synteny comparison between *Bacteriovorax* sp. As-1 and other BALO genomes was performed using MUMmer v4.0.1 [41], and synteny plots were generated with gnuplot v5.4.x.

### Taxonomic identification and phylogenetic analysis

The taxonomic position of *Bacteriovorax* sp. As-1 was assessed using genome-based digital DNA–DNA hybridization (dDDH) via the Type Strain Genome Server (TYGS) [42, 43] and average nucleotide identity (ANI) calculated with FastANI [44]. Phylogenetic relationships were primarily resolved by a core genome phylogeny generated with the UBCG2 pipeline [45], which produced a maximum-likelihood tree based on concatenated single-copy core genes with 100 bootstrap replicates. To complement this analysis, a 16S rRNA gene tree was reconstructed with TYGS, confirming the genus-level placement of the isolate.

### Detection of genomic islands and mobile genetic elements

The genomic architecture and MGEs of *Bacteriovorax* sp. As-1 were analyzed using a suite of bioinformatics tools to assess genetic mobility and identify genomic features. G + C content analysis was performed to detect sequence variations and identify potential genomic islands and HGT. Integrons were identified using Integron Finder v2.0.5 to assess potential gene integration sites [46] whereas insertion sequences (ISs) were identified using ISFinder (BLASTN 2.2.31+) with an E-value threshold of 0.05 [47]. Genomic islands (GIs) were detected using the IslandViewer 4 web server, which integrates IslandPath-DIMOB and SIGI-HMM prediction methods [48]. Prophage regions were predicted using PHASTER to identify potential bacteriophage integration sites and characterize their genomic features [49]. CRISPR arrays and associated genes were identified using CRISPRCas Finder to confirm the presence of CRISPR-Cas systems [50].

### Identification of antimicrobial resistance genes

ARGs were identified using the Comprehensive Antibiotic Resistance Database (CARD) [51] and AMRFinderPlus v3.12.8 [52], with all candidate hits manually validated through InterProScan [53]. To determine whether the ARGs detected in *Bacteriovorax* sp. As-1 were unique to this strain or also present in related predators, comparative screening was performed across five closely related *Bdellovibrio*-and-like organism (BALO) genomes (Table 1).

**Table 1:** BALO strain information, representative prey, and antibiotic resistance gene (ARG) profiles.

**Bacterial species**	**Strain**	**Isolation**	**Representative prey** *	**Antibiotic resistance genes (ARGs)**		**References**
		**Source**		**No.**	**Gene list**	
*Bacteriovorax* sp.	As-1	Rainbow fecal	*A. baumannii* *A. salmonicida* *E. coli* *P. fluorescens* *Salmonella enterica* *V. anguillarum* *V. parahaemolyticus*	13	- **Tetracycline**: tetM (RPP); tetA/tetC/tetG (efflux) - **Chloramphenicol**: catB - **β-lactam**: blaOXA-18 - **Trimethoprim**: dfrA20 - **Sulfonamide**: sul2 - **Aminoglycoside**: aadA - **Efflux-associated genes (potential MDR)**: MdtK, sugE, acrB, MFS drug efflux transporter (Bcr/CmlA subfamily)	[54–56] [57] [58] [59] [60] [61] [62, 63]
*Bacteriovorax stolpii*	DSM 12778^T^	Unknown	Identical to that of ***Bacteriovorax*** sp. As-1.	5	- **Tetracycline**: tetA - **Efflux-associated genes**: mdtC, mdtB, mdtK, MFS drug efflux transporter (Bcr/CmlA subfamily)	[56] [62–65]
*Bacteriovorax stolpii*	AC01	Snail	Unknown	8	- **Tetracycline**: tetA **- Efflux-associated genes (potential MDR)**: mdtC, sugE, mdtB, mdtK, emrB, mdtA, MFS drug efflux transporter (Bcr/CmlA subfamily)	[56] [62–65]
*Peredibacter starrii*	A3.12^T^	Soil	*Pseudomonas* sp.	7	- **Efflux-associated genes (potential MDR)**: emrB, acrA, acrB, emrA, mdtK, sugE, MFS drug efflux transporter (Bcr/CmlA subfamily)	[62, 63]
*Halobacteriovorax marinus*	SJ^T^	Seawater	*V. parahaemolyticus*	5	- **Efflux-associated genes (potential MDR)**: mdtK, sugE, acrB, mdtA, MFS drug efflux transporter (Bcr/CmlA subfamily)	[62, 63]
*Halobacteriovorax vibrionivorans*	BL9^T^	Seawater	*V. alginolyticus*	3	- **Efflux-associated genes (potential MDR)**: acrB, mdtA, MFS drug efflux transporter (Bcr/CmlA subfamily)	[62, 65]

^*^Entries for *Bacteriovorax* sp. As-1 and ***Bx. stolpii*** DSM 12778^T^ were **tested in this study** (*n* = 3; see Fig. 2A). Entries for other strains are **summarized from the literature** (see References column).

### Temperature and prey spectrum assays

Fresh predatory cultures of *Bacteriovorax* sp. As-1 and *Bx. stolpii* DSM 12778^T^ were grown as described above. They were then spotted (10 μl) on top agar plates prepared with *A. salmonicida* BB21151NE. The plates were incubated at different temperatures and the spot plaque sizes were measured daily. The same basic protocol was used to determine the *Bacteriovorax* sp. As-1 and *Bx. stolpii* DSM 12778^T^ prey spectra. For this, the top agar plates were prepared using *A. salmonicida* or one of several other potential prey species. The predator was then spotted (10 μl) on the top agar plates and incubated at 30°C. Plaque formation was assessed at 48 and 72 h.

### Predatory activity and titer burst assays

Overnight *A. salmonicida* prey cultures were harvested by centrifugation (4200 g, 10 min), washed with sterilized HEPES buffer and resuspended to an OD of 1.0. *Bacteriovorax* sp. As-1 cultures were grown overnight as described above and diluted (1:100 (v:v)) directly into the prepared prey culture, giving an initial predator-to-prey ratio (PPR) value of ~0.03. The flasks were then incubated at 30°C with shaking at 250 rpm. At set times, samples were taken to measure the prey (colony forming units (CFUs)) or predator (plaque forming units (PFUs)) viabilities.

### Scanning electron microscopy and confocal imaging

The morphology and predatory behavior of *Bacteriovorax* sp. As-1 were observed using scanning electron microscopy (SEM) and confocal microscopy, respectively, following the protocols described previously [15]. Briefly, the samples were prepared by pelleting the overnight culture of *A. salmonicida* and washing them twice with HEPES buffer before adjusting the initial OD to 4.0. This was then mixed at a 1:1 (v:v) ratio with a 10-fold concentrated culture of *Bacteriovorax* sp. As-1. The cultures were then incubated at 30°C with shaking at 250 rpm. Samples were taken at designated time points and fixed by mixing them 1:1 (v:v) with 5% glutaraldehyde and 8% paraformaldehyde. After an hour at room temperature, the samples were stored at 4°C.

For SEM, the fixed samples were loaded onto silicon wafers and allowed to settle for an hour before aspirating away the liquid and washing the wafers for 10 min with HEPES buffer. The wafers were then immersed in a 1% osmium tetroxide solution for 90 min and subsequently washed twice more for 10 min each in HEPES buffer. The samples were dehydrated by sequential immersion in 25%, 50%, 70%, 90%, and finally twice in 100% ethanol, with each step lasting 10 min. The samples were completely dried in a 60°C oven overnight, after which the wafer was attached to a holder using carbon tape. After coating the samples with platinum via sputtering at 20 mA for 3 min, they were imaged under Hitachi SU8220 SEM.

For fluorescent *in situ* hybridization (FISH), the test was performed as described previously [66]. Briefly, the fixed samples were washed twice by centrifugation (16 000 × g, 3 min) and resuspended in hybridization buffer (0.9 M NaCl, 20 mM Tris–HCl, 0.1% SDS, 35% formamide) carrying a Cy3-labeled probe (5′-CAC CCT TCG TAT TAC CGC-3′) targeting the 16S rRNA gene of *Bacteriovorax*. The samples were incubated at 46°C for 2 h, after which the bacterial cells were pelleted (16 000 × g, 3 min) and washed with wash buffer (20 mM Tris–HCl, 0.1% SDS, 5 mM EDTA). After incubation at 48°C for 20 min in the dark, the samples were imaged using a confocal microscope (LSM780, Carl Zeiss).

### Antibiotic resistance profile for *Bacteriovorax* sp. As-1

The *Bacteriovorax* sp. As-1 and *Bx. stolpii* DSM 12778^T^ antibiotic resistance profiles were assessed following a previously published protocol [67] with slight modification. Briefly, a fresh plaque of the predator was grown in liquid culture overnight as described above. In the wells of 96-well plates (SPL, Korea), the antibiotics were serially diluted in 100 μl HEPES buffer (25 mM (pH 7.2) with 3 mM MgCl_2_ and 2 mM CaCl_2_). The controls had no antibiotic present. Tests were performed using two prey strains (*A. salmonicida* and *Escherichia coli* DSM 613) independently. To the wells, 80 μl of the prey bacterium (prepared to an OD_600nm_ of 2.5 in sterile HEPES buffer) and 20 μl of a 10-fold diluted predatory culture were added. The plates were then incubated at 30°C. The OD_600nm_ of each well was then measured after 24 h and used to determine the minimum inhibitory concentration (MIC) for this predator and *Bx. stolpii* DSM 12778^T^.

### Horizontal gene transfer assays

The potential for *Bacteriovorax* sp. As-1 to serve as a donor of ARGs to other microbes was explored using *Acinetobacter baylyi* ADP-1. This microbe was selected as the recipient because it is both naturally competent [68] and not a prey for *Bacteriovorax* sp. As-1. The MICs for this bacterium in cation-adjusted MHB (CA-MHB) and lysogeny broth (LB) were 6.25 mg/l SDZ/TMP in CA-MHB and LB, 0.78 mg/l tetracycline in CA-MHB and LB and 3.13 mg/l chloramphenicol in CA-MHB and 6.25 mg/l chloramphenicol in LB.

The tests were performed using DNB top agar prepared on NB bottom agar. The top agar was prepared with *A. salmonicida* (OD 1.0) and *A. baylyi* ADP-1 (OD 0.02). The individual antibiotics were also added to the top agar to achieve a concentration of 0.5×, 1×, and 2× based on the measured CA-MHB MICs for *A. baylyi* ADP-1. Control plates were also prepared where no antibiotics were added to the agar. Freshly grown *Bacteriovorax* sp. As-1 (10 μl, 0.45 μm-filtered) was spotted onto the top agar and the plates were stored at 30°C to allow predation to occur. After 2 and 4 days, regions from the plaques (~50 mg of top agar) were transferred to 15 ml conical tubes and vortexed with 3 ml of LB medium for 5 min. The cultures were then diluted 1:100 into fresh LB medium and 100 μl was spread out on LB agar plates prepared with the above antibiotics at 1×, 2×, and 4× MIC. The plates were then incubated at 37°C to allow growth of only *A. baylyi* ADP-1 as this temperature is not permissible for *A. salmonicida*. Colonies growing on the plates were streaked onto fresh LB agar plates with the same antibiotic and concentration and grown overnight once more at 37°C.

Two colonies growing on SDZ/TMP LB agar plates were isolated and grown in liquid cultures. After confirming they were *A. baylyi* ADP-1 via 16S rRNA gene sequencing, PCR was used to check for the presence of different ARGs - *dfrA20* (As-TF – GGAAACGACAACGGGTCACG and As-TR – ATCTTCTTCTTCCCATTCTCCC), *sul2* (Sul2-F – CGCTCATCATTTTCGGCATCG and Sul2-R – GCAATGTGATCCATGATGTCGC), and *catB* (catB-F – GAACCCCAATATTATTGTGGGG and catB-R – GGCTTTGCAGGACTTCCACC).

### Statistical analyses

Each experiment was performed at least in triplicate and the standard deviations are presented as error bars in the graphs. To compare two data groups, statistical analyses were performed using the Student t-test. Statistically significant groups at *P* values of <.05, .01, or .001 are indicated within the figures (a, b, or c, respectively).

## Results and discussion

### Genomic features of *A. salmonicida* BB21151NE (prey strain)

**Figure 1:**
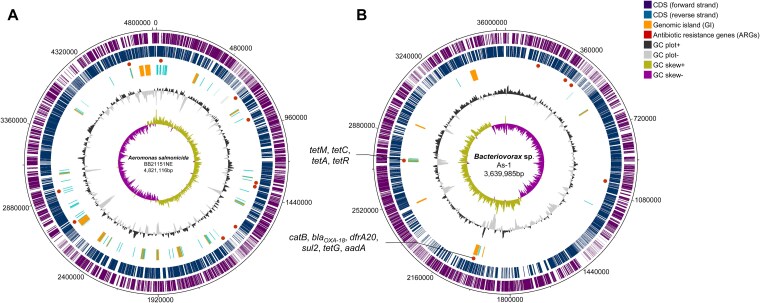
Genomic features and coverage analysis of *A. salmonicida* BB21151NE and *Bacteriovorax* sp. As-1. Circular maps of (A) *A. salmonicida* BB21151NE and (B) *Bacteriovorax* sp. As-1 showing CDS, genomic islands (GIs), ARG loci, insertion sequences (ISs), GC content, and GC skew. Full ARG lists: As-1—Table 3; Aeromonas—Table S3.

The genome of *A. salmonicida* BB21151NE was assembled into a single circular chromosome of 4 821 116 bp (110× coverage, G + C content 58.3%) along with four plasmids (Fig. 1, Table 2). The final assembly comprised five contigs (>100 bp), indicating a high degree of continuity. Quality assessment showed 99.75% completeness by CheckM and 100% completeness by BUSCO, confirming the assembly’s accuracy and taxonomic consistency (Table S1). Genome annotation predicted 4529 coding sequences, including nine ARGs, 28 rRNA genes, 116 tRNA genes, and one tmRNA, thereby providing a comprehensive overview of the genetic repertoire of this pathogenic strain (Tables 2 and S2).

**Table 2:** Summary of genomic features and assembly statistics for *Bacteriovorax* sp. As-1 and *A. salmonicida* BB21151NE.

**Attribute**	***Bacteriovorax* sp. As-1**	***A. salmonicida* BB21151NE**
**Genome assembly**		
Genome size (bp)	3 639 985	4 821 116
Genome coverage (×)	156×	110×
Number of contig (>100 bp)	1	5
GC content (%)	40.8	58.3
**Largest contig (bp)**		
N50	3 639 985	4 681 543
N90	3 639 985	4 681 543
L50	1	1
L75	1	1
**Genome annotation**		
Total CDS	3638	4529
Antibiotic Resistance Genes	13	9
tRNA	35	116
rRNA	6	28
tmRNA	1	1

### *Bacteriovorax* sp. As-1 predation activities

A recent study showed the relative abundance of *Proteobacteria*, a group to which predatory bacteria such as *Bacteriovorax* belong, in the gut microbiota of rainbow trout increased following OTC treatment [13]. The same fecal sample was used in an enrichment culture to isolate predatory bacteria exhibiting strong lytic activity against *A. salmonicida*, a fish pathogen [69–71]. A single plaque with clear edges was propagated, and this isolate’s small subunit (SSU) 16S rRNA gene sequence (NCBI Accession PV258608) showed 99.7% identity to that of *Bx. stolpii* DSM 12778^T^; we designated it *Bacteriovorax* sp. As-1.

**Figure 2:**
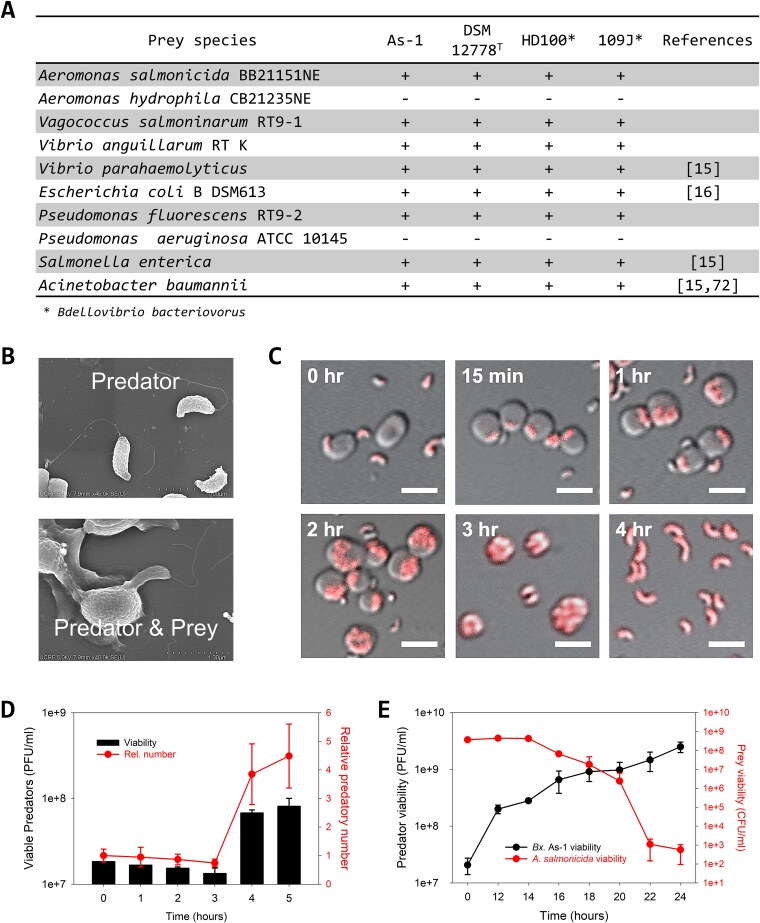
*Bacteriovorax* sp. As-1 predation activities. (A) Prey spectra for *Bacteriovorax* sp. As-1 and *Bx. stolpii* DSM12778^T^ under the same conditions are identical. (*n* = 3) (B) Scanning electron microscopic images of *Bacteriovorax* sp. As-1 alone and during predation showing attachment to *A. salmonicida*. (C) Confocal microscopic images of fluorescently labelled *Bacteriovorax* sp. As-1 obtained at different time points during predation on *A. salmonicida*. Scale bar, 2 μm. (D) Titer burst assay showing *Bacteriovorax* sp. As-1 viability increase over time during predation on *A. salmonicida*, showing release of the progeny after 4 h (PPR = 0.03; *n* = 3). (E) Predation kinetics between 0 and 24 h. The *A. salmonicida* viabilities reduced by nearly 6-log (from 4.5 × 10^8^ CFU ml^−1^ to 567 CFU ml-^1^) as the predator densities increased 2-log, reaching a maximum of 2.5 × 10^9^ PFU ml^−1^. (*n* = 3).

We found *Bacteriovorax* sp. As-1 predatory activities closely mirrored those of *Bx. stolpii* DSM 12778^T^ as their optimum growth temperature (30°C) and prey spectrum were identical (Fig. 2A) [72]. Similar to other BALO isolates, *Bacteriovorax* sp. As-1 was a short vibroid cell possessing a single polar flagellum (Fig. 2B). Moreover, consistent with the known biology of BALO strains, *Bacteriovorax* sp. As-1 attaches to its prey (*A. salmonicida*) and penetrates into its periplasm, where it grew, septated, and then lysed the bdelloplast after ~4 h (Fig. 2C). This is further supported by the titer burst, the time when the prey bdelloplast lysed and the predatory progeny within were released, which occurred around 4 h (Fig. 2D) and the average number of *Bacteriovorax* sp. As-1 progeny was four, a value i.e. fairly typical for BALOs [15, 73, 74]. The *A. salmonicida* viabilities reduced nearly 6-log over a 24-h period due to predation, from 4.5 × 10^8^ CFU ml^−1^ to just 567 CFU ml^−1^ (Fig. 2E). During the same period, the predator density increased by 2-log, reaching a maximum of 2.5 × 10^9^ (plaque-forming units) PFU ml^−1^, a value i.e. similar with other predatory strains, including *B. svalbardensis* [15] and *Bdellovibrio bacteriovorus* HD100 [75]. Collectively, these findings suggest *Bacteriovorax* sp. As-1 shares key phenotypic traits with *Bx. stolpii* DSM 12778^T^.

### Horizontal Acquisition of Antibiotic Resistance Genes in *Bacteriovorax* sp. As-1

**Figure 3:**
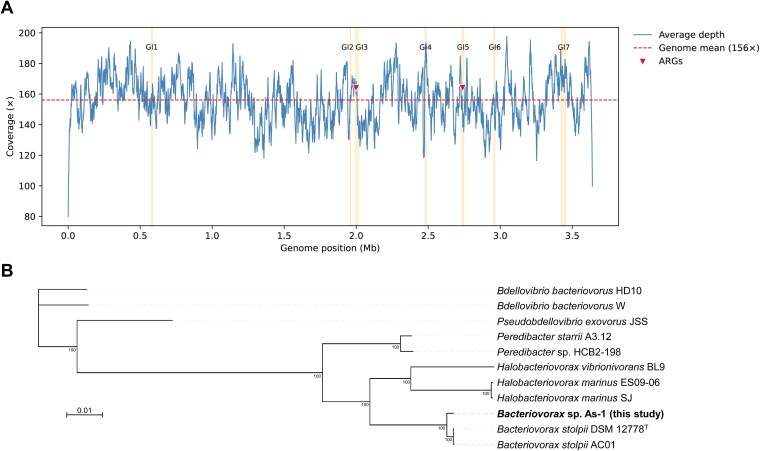
Genome-wide coverage consistency and phylogenetic placement of *Bacteriovorax* sp. As-1. (A) ONT coverage across the As-1 chromosome (1-kb bins). Vertical bands mark GI boundaries; inverted triangles denote ARG loci; dashed line, genome-wide mean (~156×). (B) Maximum-likelihood core-gene phylogeny of *Bacteriovorax* sp. As-1 and 10 related BALO genomes reconstructed with UBCG2 (RAxML, 100 bootstrap replicates) and visualized in iTOL. Scale bar, 0.01 substitutions per site.

**Figure 4:**
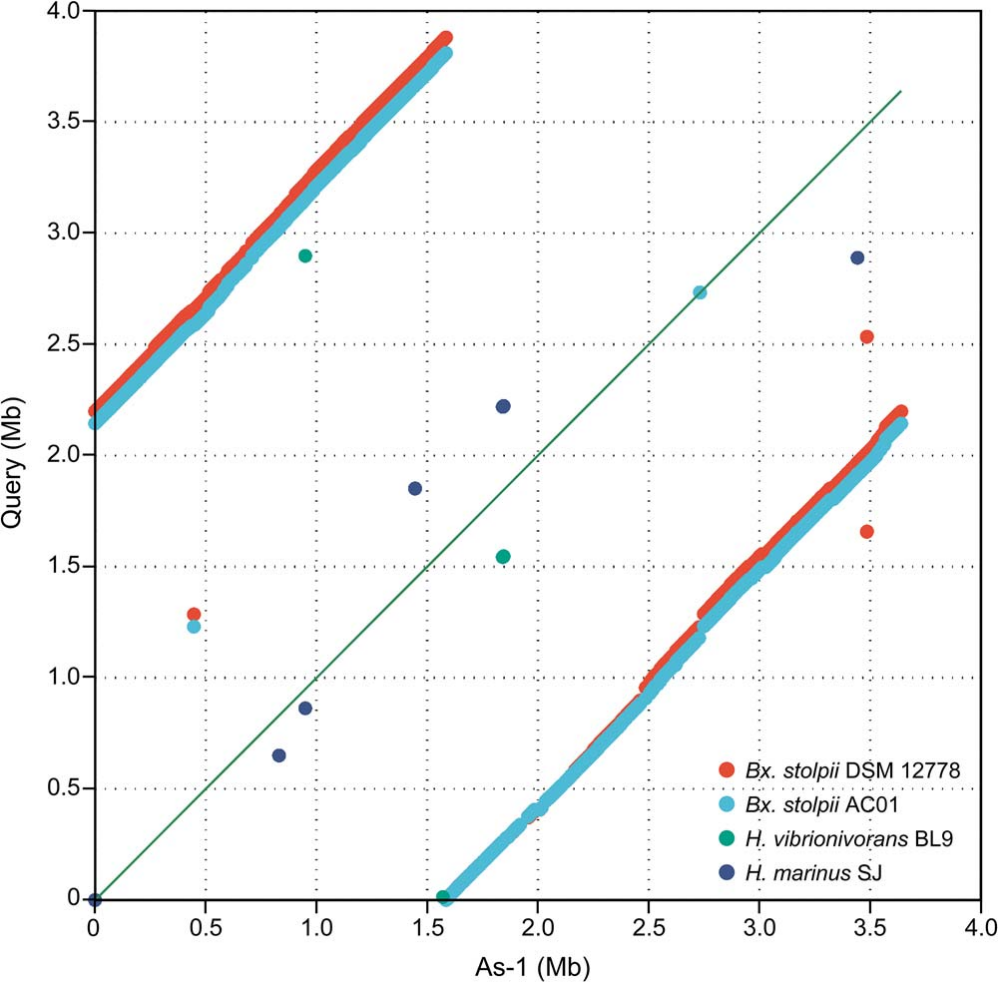
Whole-genome synteny of *Bacteriovorax* sp. As-1 (x-axis) with related BALOs (y-axis): *Bx. stolpii* DSM 12778^T^, *Bx. stolpii* AC01, *Halobacteriovorax vibrionivorans* BL9, *H. marinus* SJ. Dot plots were generated with MUMmer v4 (≥1 kb anchors, ≥95% identity). The diagonal indicates collinearity; off-diagonal points indicate rearrangements.

The assembled genome of *Bacteriovorax* sp. As-1 (NCBI Accession CP184341) consisted of a single-contig assembly of 3 639 985 bp with 156× coverage and a G + C content of 40.8% (Table 2). Uniform coverage depth across the As-1 chromosome was also observed (Fig. 3), supporting the robustness of the assembly and confirming the absence of structural gaps or prey-derived sequences. The genome exhibited 88.7% completeness (BUSCO) and was predicted to encode 3638 coding sequences, 13 antibiotics resistance genes, 35 tRNA genes, 1 tmRNA, and 13 insertion elements, but no integrons were identified. Phylogenetic analyses based on both 16S rRNA and core genes placed *Bacteriovorax* sp. As-1 in close proximity to *Bx. stolpii* (Fig. 3B and S1). However, its dDDH (46%) and ANI (92.6%) values (Fig. S2 and Table S3) were well below the generally accepted thresholds for species delineation (≥70% for dDDH and ≥95% for ANI), indicating that this predator may represent a new species within the genus *Bacteriovorax*. Synteny analysis supported this conclusion. Whereas large-scale syntenic blocks were conserved with *Bx. stolpii* DSM 12778 and AC01, *Bacteriovorax* sp. As-1 exhibited extensive rearrangements and unique genomic regions (Fig. 4). These patterns are consistent with the ANI and dDDH values and collectively underscore substantial genomic divergence.

The genome and 16S rRNA gene sequences for *Bacteriovorax* sp. As-1 were also analyzed using Protologger [76] to predict the ecological distribution of closely related taxa (Fig. S3). Operational taxonomic units (OTUs) from public 16S rRNA gene amplicon datasets possessing at least 97% sequence identity and 80% coverage to the 16S rRNA gene of this isolate were identified at substantial percentages in wastewater, rhizosphere, freshwater, activated sludge and soil metagenomes (25.3%, 48.1%, 32.7%, 17.2%, and 17.6%, respectively) but at low relative abundances (0.01%–0.07%) (Fig. S3). Lower detection frequencies were observed in the guts of insects and animals, with values ranging between 0 and 2%, and similarly low relative abundances (0%–0.08%).

**Figure 5:**
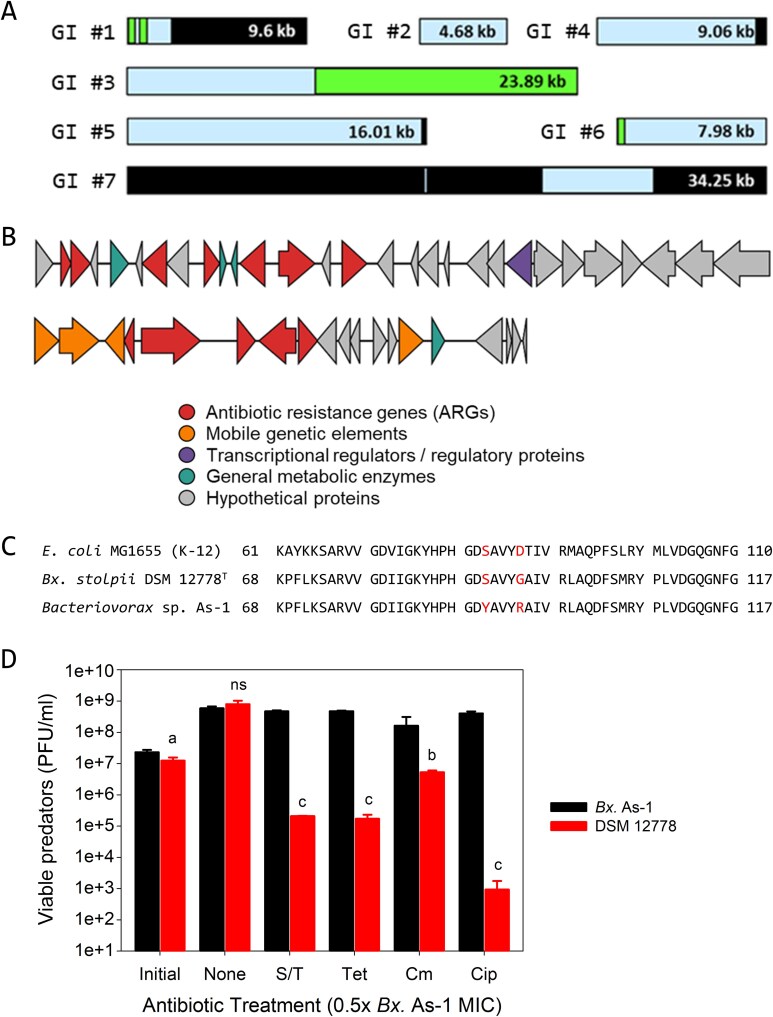
*Bacteriovorax* sp. As-1 is resistant to multiple antibiotics. (A) Homology of As-1 genomic islands (GI #1–#7) to *Bx. stolpii* DSM 12778^T^. Bars are to scale; colors indicate pairwise identity (no detectable homology, <80%, >80%). Some islands (#3, #5) contain conserved tracts, whereas others show little or no homology. (B) Gene organization of two As-1 islands (GI#3, GI#5), highlighting the presence of the ARGs within each; colors as in Fig. 1. (C) QRDR alignment of GyrA showing the S90Y substitution in As-1 (red) relative to *E. coli* MG1655 and *Bx. stolpii* DSM 12778^T^. (D) Viable predator counts (PFU ml^−1^) after 24 h at 0.5× MIC for the indicated antibiotics. As-1 maintains higher viability than DSM 12778^T^ across several treatments. Statistics between predators: a, *P* < .05; b, *P* < .01; c, *P* < .001; ns, not significant; *n* = 4.

Seven genomic islands (GIs) identified within the *Bacteriovorax* sp. As-1 genome suggested possible HGT events. Among these, two genomic islands (GIs #1 and #7) showed partial coverage and nucleotide identity values exhibiting strong homology with the *Bx. stolpii* DSM 12778 T genome (>83%; Fig. 5A), although all contained distinct regions lacking homology. Within these unique regions, 56 genes were annotated, including 30 hypothetical genes (53.6%) and nine genes (16.0%) directly associated with antibiotic resistance (Table 3). IntegronFinder detected no complete class 1/2/3 integrons with attC arrays; only isolated integrase-like ORFs without cassette arrays were observed (Table 3; Table S4). The prey genome encodes its own ARGs (*tetA*, *bcr*/*cmlA*) (Table S2), but it lacks the IS-linked composite *tet* cluster and the *blaOXA-18–catB–aadA–dfrA20–sul2–tetG* arrangement observed in *Bacteriovorax* sp. As-1 (Table 3; Fig. 5B).

**Table 3:** Predicted genomic islands in *Bacteriovorax* sp. As-1.

No.	Genomic island (GI)			Method^a^	GC (%)^b^	% Coverage	No. of genes	ARGs
	Start	End	Size (bp)			(Homology)^c^		
1	577 612	587 208	9597	IPD	39.1	85 (97.1)	8	
2	1 958 054	1 962 735	4682	SH	31.8	0 (0)	6	
3	1 993 235	2 017 121	23 887	IPD	41.6	43 (76.7)	29	*catB, bla*_OXA-18_ *, dfrA20, sul2, tetG*, *aadA*
4	2 476 986	2 486 048	9063	SH	34.6	6 (88.2)	6	
5	2 732 067	2 748 077	16 011	Int	38.4	1 (96.8)	19	*tetM*, *tetC*, *tetA*
6	2 955 019	2 962 997	7979	IPD	42.7	4 (78.8)	11	
7	3 422 312	3 456 560	34 249	IPD	40.5	83 (94)	24	

a – GI Prediction Method (IPD - IslandPath-DIMOB; SH - SIGI-HMM; Int – Integrated)

b – *Bacteriovorax* sp. As-1 average – 40.8%

c – Homology with *Bx. stolpii* DSM 12778^T^ genome sequence. The number is percent coverage and then the percent identity.

The GI #5, which was uniquely detected in *Bacteriovorax* sp. As-1 but absent in the type strain, contained a composite tetracycline resistance gene cassette-like structure consisting of *tetM* (ribosomal protection), *tetC* and *tetA* (efflux pumps), with the local regulator *tetR* (transcriptional repressor) [54, 55]. Multiple IS3 family transposases, including IS407, IS150, IS51, and the IS2 group, were detected on both sides of this *tet* cluster. Two IS407 copies (IS #7, 11) exhibited 99.9% sequence identity, strongly indicative of duplication. This arrangement resembles a composite transposon-like structure, suggesting that the *tet* genes may have been captured by IS3 elements and function as a single transferable unit (Table 3). Additionally, GI #3 contains a resistance gene cluster consisting of *catB*, *blaOXA-18*, *aadA*, *dfrA20*, *sul2*, and *tetG*, previously associated with reduced susceptibility to chloramphenicol, β-lactams, streptomycin, trimethoprim, sulfonamides, and tetracycline [59, 60, 77–79]. In contrast, GI #1 contained ISSvi1 but lacked virulence factors or ARGs. Diverse IS families within genomic islands can act as HGT delivery tools contributing to the dissemination of virulence and resistance genes. In line with this observation, the *tet*–IS configuration in GI #5 of *Bacteriovorax* sp. As-1 indicates that the acquisition of ARGs was mediated by IS-driven HGT.

Assembly robustness was further supported by uniform read coverage across all predicted genomic islands and ARG loci. CheckM analysis indicated 93.0% completeness, 1.8% contamination, and 0% strain heterogeneity, results that are comparable with other predatory strains (Table S1). Prey genome read mapping revealed only 0.43% alignment (0.29% properly paired), confirming minimal prey carryover. We also sequenced the prey genome independently, and prey-derived ARGs do not explain the loci observed in As-1. These results indicate that the ARGs identified in As-1 are not attributable to prey contamination but are consistent with acquisition via HGT. These data support the view that a natural *Bacteriovorax* isolate harbors ARG loci within IS-rich regions consistent with acquisition via HGT, highlighting potential ecological and biotechnological concerns. This knowledge also provides a basis for the safe development of BALOs as biocontrol agents.

### *Bacteriovorax* sp. As-1 is resistant to multiple antibiotics

We assessed if the genomic findings align with reduced susceptibility phenotypes across antibiotics. We found quinolone resistance-determining region (QRDR) mutations were also found in *gyrA* [80, 81]. Alterations in this critical region are known to confer resistance to fluoroquinolone antibiotics, such as ciprofloxacin, by reducing the binding affinity of the antibiotic to the DNA gyrase-DNA complex, compromising bactericidal activities of these antibiotics [82]. Whereas mutations at Ser83 are commonly associated with ciprofloxacin resistance, mutations outside this region have also been reported [83]. Alignment of the DNA gyrase subunit A amino acid sequences from *Bacteriovorax* sp. As-1 and *Bx. stolpii* DSM 12778^T^ revealed the new isolate carried an S90Y mutation within the canonical QRDR (Fig. 5C), previously associated with reduced fluoroquinolone susceptibility in *Enterobacteriaceae* [82, 83], and a G94R mutation (equivalent to D87 in *E. coli* MG1655) whose role is less well documented. These changes are therefore consistent with reduced susceptibility; the S90Y mutation is a well-documented mechanism in multiple bacteria, whereas the role of G94R remains less clear and requires further study. Because both predators carried mutations in this amino acid, the resistance profiles for *Bacteriovorax* sp. As-1 and *Bx. stolpii* DSM 12778^T^ to ciprofloxacin were mapped out in parallel with the other antibiotics.

The minimal inhibitory concentrations (MICs) for the antibiotics were determined for both predators (Table 4). Except for kanamycin, *Bx. stolpii* DSM 12778^T^ was much more sensitive than *Bacteriovorax* sp. As-1, with MICs that were 4- to 400-fold lower. This was explored further in predation and predator survival assays where the 0.5x MIC antibiotic concentrations for *Bacteriovorax* sp. As-1 were employed (Fig. 5D). Except for chloramphenicol, where growth of *Bacteriovorax* sp. As-1 was minimal, this predatory strain grew well in the presence of all the antibiotics to final densities that were similar to those of the untreated controls, indicating the chromosomal ARG repertoire is associated with reduced susceptibility under our assay conditions. In contrast, *Bx. stolpii* DSM 12778^T^ was sensitive and saw viability losses with all five antibiotics, particularly ciprofloxacin where this loss approached nearly 6-log. As *Bacteriovorax* sp. As-1 growth was not impacted by this antibiotic, these findings are consistent with reduced fluoroquinolone susceptibility associated with S90Y, a well-documented mechanism reported in multiple bacteria. However, the potential importance of the G94R mutation present in the *Bacteriovorax* sp. As-1 genome (Fig. 5C) remains to be clarified and should be studied further to determine if it provides additional resistance alongside the S90Y mutation.

**Table 4:** Antibiotic resistance profiles for *Bx. stolpii* DSM 12778^T^ and *Bacteriovorax* sp. As-1.

Antibiotic	*Bx. stolpii*	*Bacteriovorax* sp.	Fold increase
	DSM 12778^T^	As-1	
Sulfa/Trimethoprim	< 3.2 μg/ml	> 400 μg/ml	> 128
Tetracycline	< 1.6 μg/ml	25 μg/ml	> 16
Chloramphenicol	< 1.6 μg/ml	25 μg/ml	> 16
Ciprofloxacin	< 1.6 μg/ml	6.3 μg/ml	> 4
Kanamycin	6.3 μg/ml	6.3 μg/ml	1

### Ecological considerations of *Bacteriovorax* sp. As-1

The predatory mechanisms employed by BALO strains are known to inhibit the growth of many pathogens, with several studies also highlighting their ability to hydrolyze their prey’s genomic and plasmid DNA, including the ARGs present [22, 84]. This suggests a direct role for predatory bacteria in reducing AMR. However, a recent study revealed other predatory bacteria, such as *Myxococcus xanthus*, can indirectly enrich antibiotic-resistant bacteria in the environment by releasing toxins or secondary metabolites during mass lysis induced by nutrient starvation [85]. Within this broader context of complex predatory-prey interactions, our data for *Bacteriovorax* sp. As-1 suggest that this predator acquired ARGs through HGT, presumably in the gut of rainbow trout. This implies that BALOs, in addition to their role in eliminating ARGs from the environment, can also become potential reservoirs of AMR by harboring ARGs themselves, thereby emphasizing the multidimensional and intricate impact of predatory bacteria on the AMR ecosystem. Given the high bacterial densities in this environment, conjugation is a plausible hypothesis [86–89], but no *tra*/*mob* genes were detected and transfer was not demonstrated; accordingly, we treat these as putative non-mobilizable integrative regions. To clarify this, further studies will be necessary to determine the mobility of these integrative elements and the potential for ARG transfer in BALOs.

In this study, *Bacteriovorax* sp. As-1 was isolated from an OTC-exposed gut environment. Antibiotic selective pressures not only shift the relative abundance of resistance genes within microbial communities but also promote the horizontal transfer of ARGs via MGEs, such as plasmids and insertion elements [90–92]. It was also reported that antibiotic treatment significantly increased the relative abundance of ARGs in fish feces [13]. Specifically, OTC treatment elevated *sul2* abundance by more than sevenfold and that of *tetB* and *tetM* by up to tenfold. These genes were identified in various bacterial genera, particularly *Flavobacterium* and *Pseudomonas*, suggesting that HGT frequently occurs among microorganisms under antibiotic exposure [93–97]. Additionally, in the SDZ/TMP-treated groups, the relative abundance of *sul* and *dfr* genes increased and showed a strong positive correlation with *intI1*. IntI1 functions as an integrase and recombination element and, during antibiotic exposure, may serve as a key factor promoting the dissemination of ARGs via HGT [98–100]. These findings suggest the antibiotic-induced selective pressures within the rainbow trout gut facilitated ARG acquisition in *Bacteriovorax* sp. As-1, leading to their stable integration into its genome, contributing to the persistence of this predator.

Nearly all of the GIs identified in *Bacteriovorax* sp. As-1 were genetic elements absent in the *Bx. stolpii* DSM 12778^T^, except for minor fragments (Fig. 5A). These patterns are consistent with HGT-mediated acquisition of ARGs. Moreover, certain IS3 family transposases (IS407, IS150, IS51, and the IS2 group) were found within GI #5, which harbored the OTC resistance-associated cluster (*tetM, tetC, tetA*, adjacent regulator *tetR*), whereas 10 out of the 13 IS elements were found either within or adjacent to the GIs, indicating they likely played a crucial role in the acquisition and genomic integration of the ARGs (Table S4). IS elements are known to enhance genome plasticity by mediating gene acquisition, deletion, and recombination [101, 102] and the results of our present study are consistent with this knowledge, namely that HGT processes can mediate the simultaneous acquisition of multiple genes, contributing to bacterial adaptation and genome evolution [103]. The presence of these GIs and IS elements, which constitute ~2.9% of the total genome, may have influenced the ANI and dDDH results comparing *Bacteriovorax* sp. As-1 and *Bx. stolpii* DSM 12778^T^. The ANI value of *Bacteriovorax* sp. As-1 (92.6%) was below the species delineation threshold (95%), whereas the dDDH value (46%) was also lower than the cutoff (70%). In addition, synteny analysis revealed marked structural differences even from its closest relatives. Taken together, the ANI, dDDH, and synteny results consistently suggest that *Bacteriovorax* sp. As-1 represents a potential new species within the genus *Bacteriovorax*.

The presence of these GIs and IS elements and their associated ARGs also have far-reaching ecological implications. BALOs have been explored as potential biocontrol agents in aquaculture to mitigate bacterial pathogens and their infections [14, 20, 71], offering an alternative to conventional antibiotics [22]. However, the demonstrated presence of predicted ARGs within a BALO genome raises concerns about its role in ARG dissemination. In particular, if such a strain interacts with other bacteria in the environment, a risk exists that these resistance genes may be transferred to other microbes, potentially exacerbating antibiotic resistance in the given microbial communities. Although tests performed here to evaluate this possibility with *Bacteriovorax* sp. As-1 using the naturally competent *A. baylyi* ADP-1 [68] did not find HGT (Fig. S4), the clear presence of ARGs within the genome of this predator highlights the need for natural BALO isolates to be screened for ARGs and phenotypes before environmental use. An additional concern related with the emergence of antibiotic-resistant BALOs is their downstream impacts on microbial ecosystem dynamics. Resistant BALOs may gain a selective advantage in antibiotic-rich environments, allowing them to persist and potentially outcompete other susceptible predatory strains. This bottleneck could narrow the predatory spectrum and potentially reshape microbial community structures. These findings highlight the critical need for rigorous genomic screening of new BALO isolates, particularly those from antibiotic-exposed environments, prior to their use as biocontrol agents.

## Supplementary Material

Supplemental_Information_R2_NE_wraf245

## Data Availability

All data are available in the main text or the supplementary materials.
