# Supplementary material for: Acquisition of novel antibiotic resistance genes by the bacterial predator Bacteriovorax sp. As-1
Source: ISME J. 2025 Nov 3;19(1):wraf245. doi: 10.1093/ismejo/wraf245 (PMC12642874; doi:10.1093/ismejo/wraf245)
Supplement: Supplemental_Information_R2_NE_wraf245 [file supplemental_information_r2_ne_wraf245.docx]

**Supplemental Information**

**for**

**Acquisition of Novel Antibiotic Resistance Genes by the Bacterial Predator *Bacteriovorax* sp. As-1**

**Running Title: Multidrug-resistance in a BALO isolate**

**Fathrinah Binti Kohadie^1,†^, Young-Ung Heo^2,†^, Wonsik Mun^1^, Sumin Choi^1^, Sinseong Park^1^, Yoonhang Lee^3^, Do-Hyung Kim^2,*^, and Robert J. Mitchell^1,*^**

^1^ – Department of Biological Sciences, Ulsan National Institute of Science and Technology (UNIST), Ulsan, Republic of Korea

^2^ - Department of Aquatic Life Medicine, Pukyong National University, Busan, Republic of Korea

^3^ - Department of Aquatic Life Medicine, Chonnam National University, Yeosu, Republic of Korea

^†^ - These authors contributed equally

*Correspondence:

Do-Hyung Kim - [dhkim@pknu.ac.kr](mailto:dhkim@pknu.ac.kr)

Robert J. Mitchell – [esgott@unist.ac.kr](mailto:esgott@unist.ac.kr)

**Supplemental Tables**

**Table S1.** Genome assembly completeness, sequencing quality, and prey mapping analysis of *Bacteriovorax* sp. As-1 and representative reference strains, based on CheckM, BUSCO, and genome-to-prey read mapping analyses.

| **Category** | ***Bacteriovorax* sp.** | ***Bacteriovorax* *stolpii*** | | ***Aeromonas salmonicida* subsp*. salmonicida*** |
| --- | --- | --- | --- | --- |
|  | **As-1** | **DSM 12778^T^** | **AC01** | **BB21151NE** |
|  | **(This study)** |  |  |  |
| **CheckM** |  |  |  |  |
| Completeness | 93.01% | 93.30% | 92.41% | 99.75% |
| Contamination | 1.79% | 1.79% | 1.79% | 0.42% |
| Strain heterogeneity | 0% | 0% | 0% | 0% |
|  |  |  |  |  |
| **BUSCO** |  |  |  |  |
| Complete | 88.70% | 87.10% | 87.10% | 100.00% |
|  |  |  |  |  |
| **Genome mapping** |  |  |  |  |
| mapped (to prey read) | 0.43% |  |  |  |
| properly paired | 0.29% |  |  |  |
| singletons | 0.14% |  |  |  |

Table S2. Antibiotic resistance genes identified in the genome of Aeromonas salmonicida subsp. salmonicida BB21151NE.

| **Drug class** | **Gene** | **Resistance mechanism** | **References** |
| --- | --- | --- | --- |
| β-lactam | class D β-lactamase (OXA-like) | Enzymatic inactivation (β-lactam hydrolysis, Ambler class D) | [1] |
|  | AmpC family β-lactamase | Enzymatic inactivation (β-lactam hydrolysis, Ambler class C) | [2] |
|  | Metallo-β-lactamase (*bla*_B2_CphA-like) | Enzymatic inactivation (carbapenem hydrolysis, Ambler class B2) | [3] |
| Tetracycline | *tetA* | Efflux-mediated tetracycline resistance (MFS family) | [4] |
| Efflux-associated genes (potential MDR) | RND Acriflavin (AcrB-like) | RND efflux pump, multidrug export | [5] |
|  | RND MdtF-like | RND efflux pump, multidrug export |  |
|  | RND, Acriflavin resistance protein (MdtF-like) | RND efflux pump, acriflavin resistance |  |
|  | Bcr/CmlA | MFS efflux pump, chloramphenicol/phenicol efflux | [6] |
|  | MFS efflux pump (Bcr/CmlA subfamily) | MFS efflux pump, chloramphenicol/phenicol efflux | [7] |
|  | MacB efflux pump | ABC transporter efflux pump, macrolide resistance | [8] |

**Table S3.** Average nucleotide identity (ANIm) and digital DNA-DNA hybridization (dDDH) values for *Bacteriovorax* sp. As-1 and other bacterial strains.

| **Species** | **ANI (%)** | **dDDH** |
| --- | --- | --- |
|  |  | **(d4, %)** |
| *Bacteriovorax stolpii* DSM 12778 | 92.60 | 46.0 |
| *Vibrio sinensis* BEI233 | 64.90 | 39.9 |
| *Actinobacillus minor* NM305 | 64.98 | 33.0 |
| *Frischella perrara* DSM 104328 | 64.18 | 29.5 |
| *Enterococcus xiangfangensis* NCIMB 14834 | 64.28 | 27.3 |
| *Desulfoluna butyratoxydans* MSL71 | 62.72 | 26.1 |
| *Geobacillus thermopakistaniensis* MAS1 | 62.79 | 24.0 |
| *Providencia stuartii* NCTC 11800 | 63.77 | 22.4 |
| *Escherichia fergusonii* ATCC 35469 | 63.52 | 22.4 |
| *Desulfuromonas thiophila* DSM 8987 | 63.20 | 21.1 |
| *Geobacter grbiciae* DSM 13689 | 62.91 | 21.1 |
| *Geomesophilobacter sediminis* Red875T | 63.52 | 21.1 |
| *Desulfogranum mediterraneum* DSM 13871 | 62.53 | 20.7 |
| *Pseudobacteriovorax antillogorgiicola* RKEM611 | 63.21 | 19.8 |
| *Halobacteriovorax vibrionivorans* BL9 | 66.20 | 19.1 |
| *Poseidonocella pacifica* DSM 29316 | 63.54 | 18.8 |
| *Peredibacter starrii* A3.12 | 66.10 | 18.5 |
| *Halobacteriovorax marinus* SJ | 66.01 | 18.4 |
| *Acinetobacter albensis* ANC 4874 | 63.38 | 18.3 |

**Table S4.** Predicted Insertion sequence (IS) elements in *Bacteriovorax* sp. As-1 using ISfinder**.**

| **No.** | **Insertion sequence (IS) elements** | | | | | |
| --- | --- | --- | --- | --- | --- | --- |
|  | **IS element** | **IS Family** | **Group** | **Start** | **End** | **Size** |
|  |  |  |  |  |  | **(bp)** |
| 1 | Putative IS3 family transposase ISDet3 | IS3 | IS407 | 445590 | 446405 | 816 |
| 2 | Putative IS3 family transposase ISPae1 | IS3 | IS407 | 446456 | 446725 | 270 |
| 3 | Putative IS481 family transposase ISSvi1 | IS481 |  | 578887 | 579792 | 906 |
| 4 | Putative IS1595 family transposase ISCp1 | IS256 |  | 1173242 | 1174108 | 867 |
| 5 | Putative ISL3 family transposase ISLpn11 | ISL3 |  | 1984533 | 1985732 | 1200 |
| 6 | Putative IS3 family transposase ISNmu3 | IS3 | IS2 | 1989843 | 1990676 | 834 |
| 7 | Putative IS3 family transposase ISDsp2 | IS3 | IS407 | 2732067 | 2732855 | 789 |
| 8 | Putative IS91 family transposase ISHaha4 | IS3 | IS150 | 2732867 | 2734162 | 1296 |
| 9 | IS3 family transposase ISAba2 | IS3 | IS51 | 2734352 | 2734975 | 624 |
| 10 | IS3 family transposase ISAba66 | IS3 | IS51 | 2734975 | 2735292 | 318 |
| 11 | Putative IS3 family transposase ISDsp2 | IS3 | IS407 | 2743934 | 2744722 | 789 |
| 12 | Putative ISL3 family transposase ISNmu3 | IS3 | IS2 | 2751787 | 2752620 | 834 |
| 13 | Putative IS481 family transposase ISSvi1 | IS481 |  | 3330013 | 3330918 | 906 |

**Supplemental Figures**

**Figure S1.** Phylogenetic tree based on the 16S rRNA gene sequences available in the Type Strain Genome Server (TYGS) [9]. The results show the close proximity and relation of *Bacteriovorax* sp. As-1 with *Bx. stolpii* DSM 12778^T^.

**
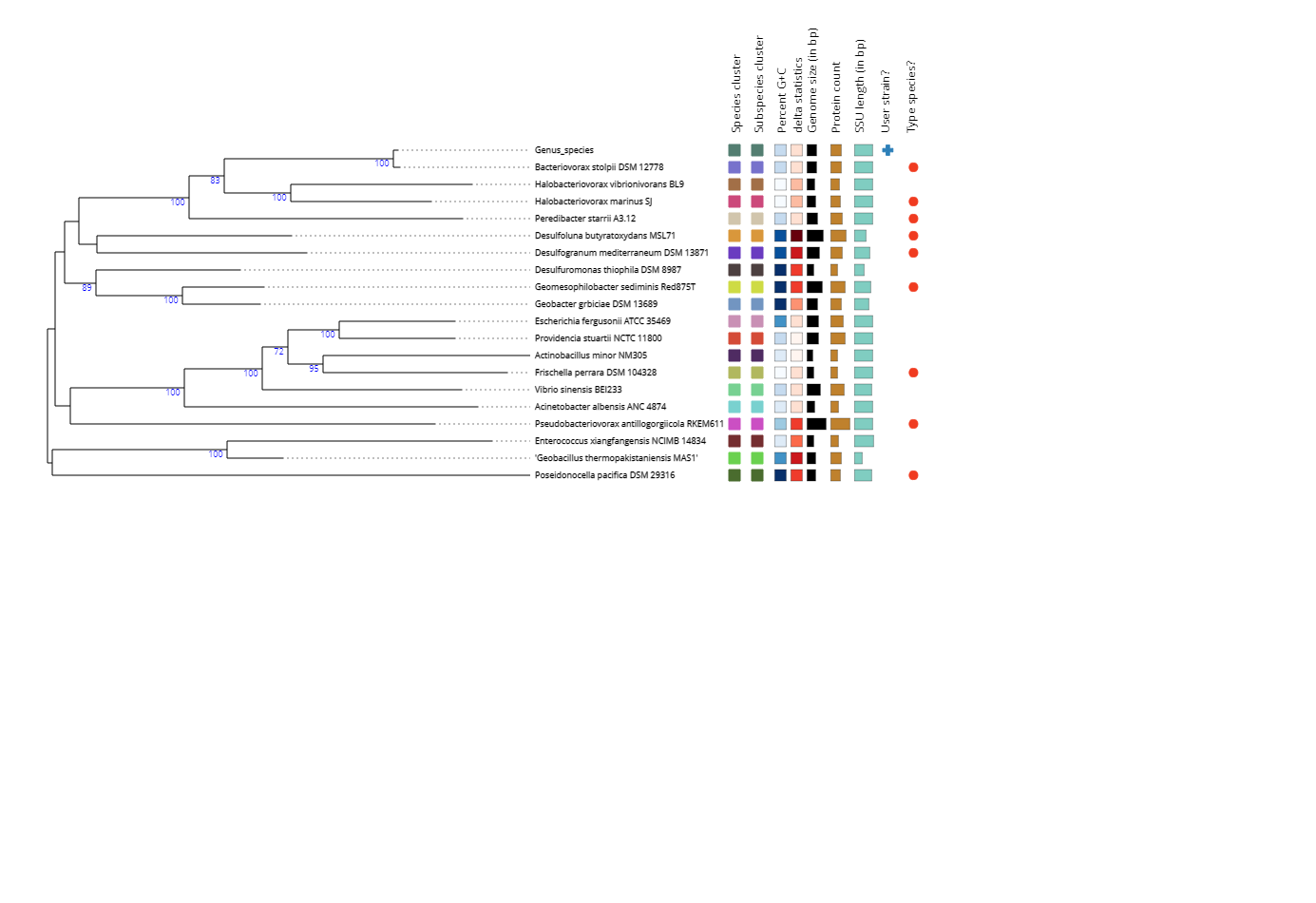
**

**Figure S2.** Average nucleotide identity (ANI) and digital DNA–DNA hybridization (dDDH) analyses of *Bacteriovorax* sp. As-1 and related strains (11 genomes). (A) ANI values were calculated using fastANI [10]. (B) dDDH values were estimated using TYGS [9]. The color scale indicates percentage similarity, with higher values shown in red.

**
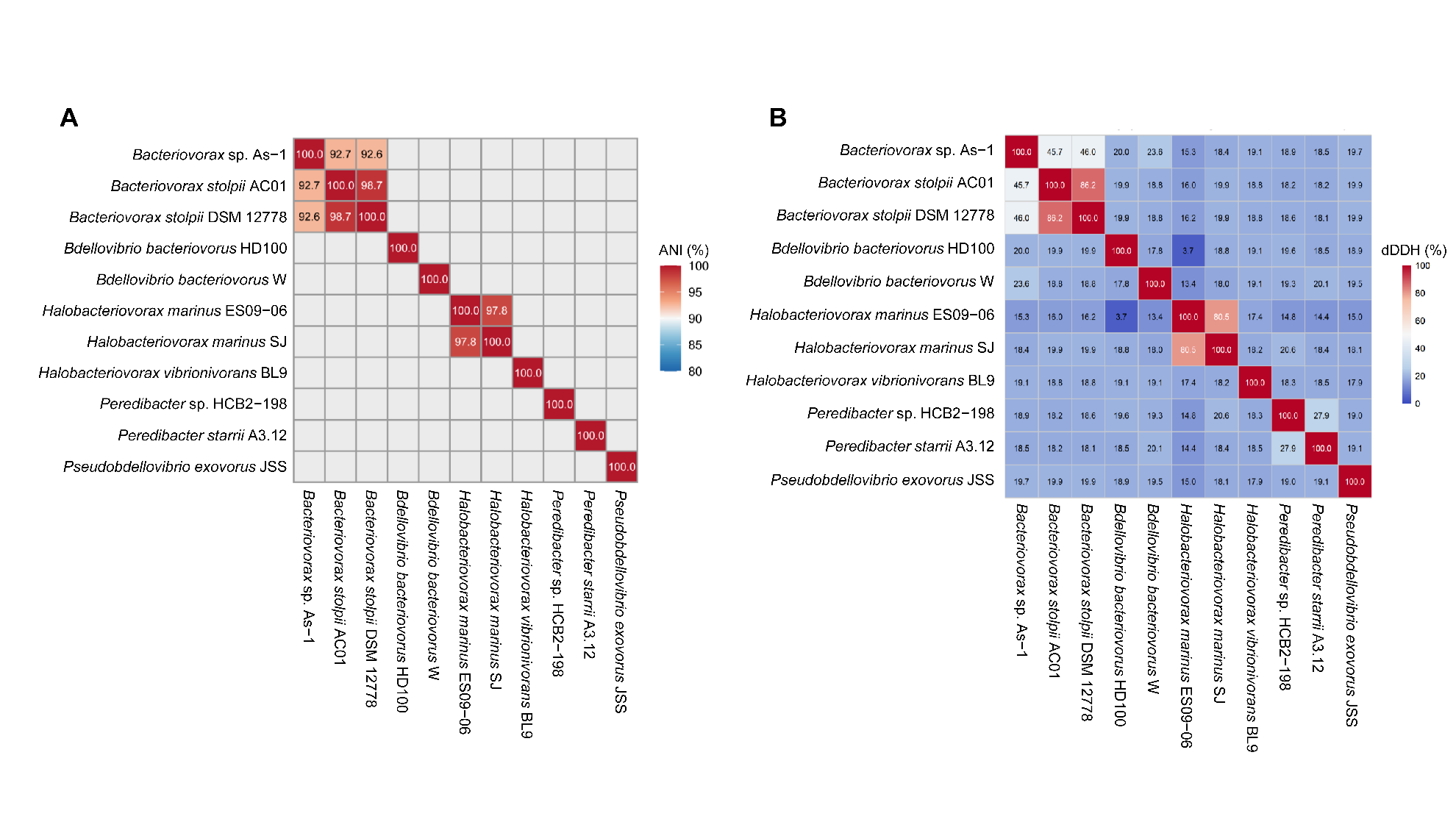
**

**Figure S3.** Spider plot of a Protologger 16S rRNA gene hit analysis [11]. The results show the ecological distribution and habitat preference for species showing at least 97% sequence identity and 80% coverage to the 16S rRNA gene of *Bacteriovorax* sp. As-1.

**
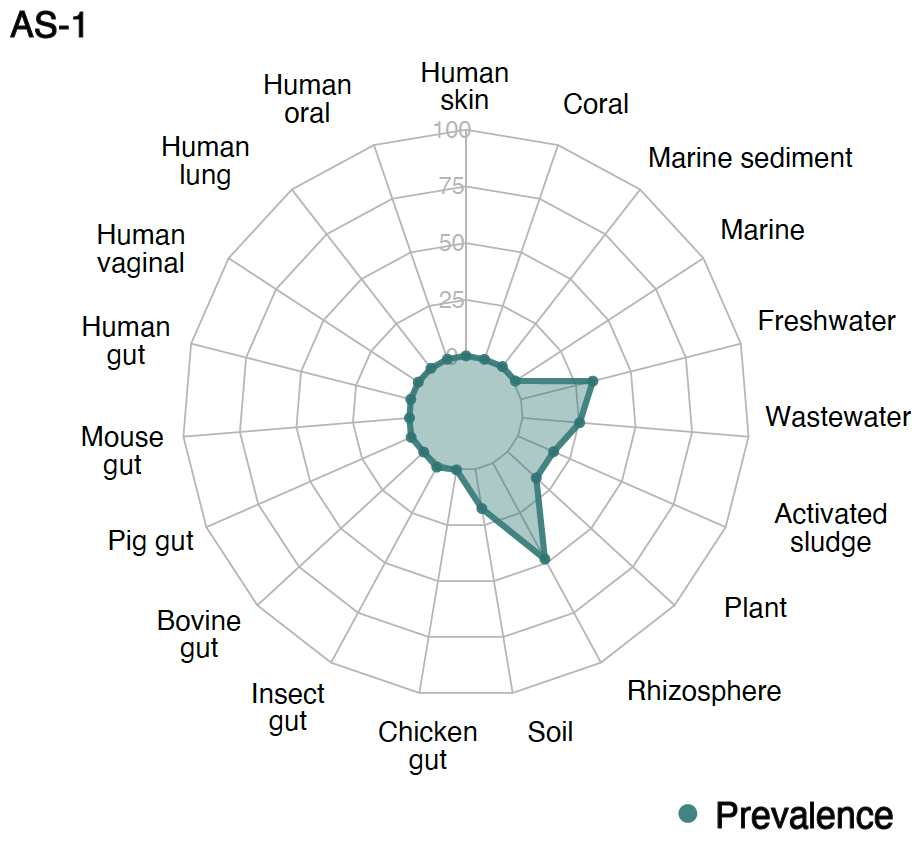
**

**Figure S4.** PCR results showing the presence of the *catB*, *dfrA20* and *sul2* genes within *Bacteriovorax* sp. As-1 but their absence in *A. baylyi* ADP-1 and two of its clones showing slight resistance (4x MIC) to SDZ/TMP. These results show no apparent HGT from this predator to *A. baylyi* ADP-1 under the conditions tested. Lanes: Std – standards; 1 – *Bacteriovorax* sp. As-1; 2 - *A. baylyi* ADP-1; 3 - *A. baylyi* ADP-1 clone #1; 4 - *A. baylyi* ADP-1 clone #2; 5 - *A. salmonicida* BB21151NE.

**
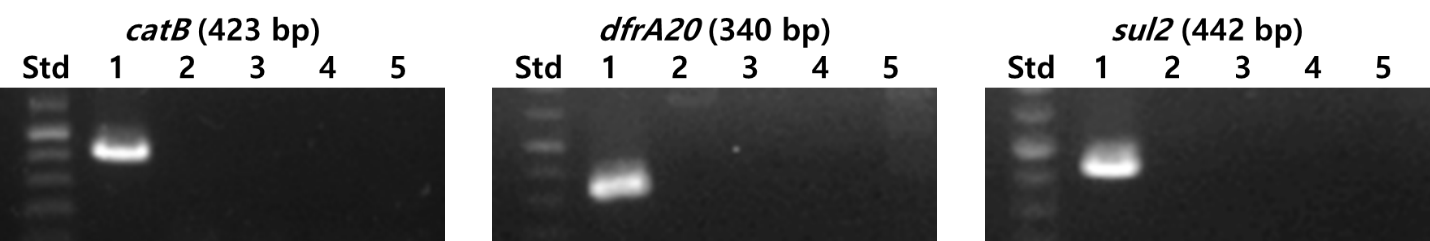
**

**References**

1. Oliva M, Dideberg O, Field MJ. Understanding the acylation mechanisms of active-site serine penicillin-recognizing proteins: A molecular dynamics simulation study. *Proteins*. 2003;**53**:88–100 https://doi.org/10.1002/prot.10450

2. Preston KE, Radomski CC, Venezia RA. Nucleotide sequence of the chromosomal ampc gene of enterobacter aerogenes. *Antimicrob Agents Chemother*. 2000;**44**:3158–62 https://doi.org/10.1128/AAC.44.11.3158-3162.2000

3. Yong D, Toleman MA, Giske CG *et al.* Characterization of a new metallo-beta-lactamase gene, bla(ndm-1), and a novel erythromycin esterase gene carried on a unique genetic structure in klebsiella pneumoniae sequence type 14 from india. *Antimicrob Agents Chemother*. 2009;**53**:5046–54 https://doi.org/10.1128/AAC.00774-09

4. Moller TS, Overgaard M, Nielsen SS *et al.* Relation between tetr and teta expression in tetracycline resistant escherichia coli. *BMC Microbiol*. 2016;**16**:39 https://doi.org/10.1186/s12866-016-0649-z

5. Ma D, Cook DN, Alberti M *et al.* Molecular cloning and characterization of acra and acre genes of escherichia coli. *J Bacteriol*. 1993;**175**:6299–313 https://doi.org/10.1128/jb.175.19.6299-6313.1993

6. Bentley J, Hyatt LS, Ainley K *et al.* Cloning and sequence analysis of an escherichia coli gene conferring bicyclomycin resistance. *Gene*. 1993;**127**:117–20 https://doi.org/10.1016/0378-1119(93)90625-d

7. Nishino K, Yamasaki S, Nakashima R *et al.* Function and inhibitory mechanisms of multidrug efflux pumps. *Front Microbiol*. 2021;**12**:737288 https://doi.org/10.3389/fmicb.2021.737288

8. Kobayashi N, Nishino K, Yamaguchi A. Novel macrolide-specific abc-type efflux transporter in escherichia coli. *J Bacteriol*. 2001;**183**:5639–44 https://doi.org/10.1128/JB.183.19.5639-5644.2001

9. Meier-Kolthoff JP, Göker M. Tygs is an automated high-throughput platform for state-of-the-art genome-based taxonomy. *Nat Commun*. 2019;**10** https://doi.org/10.1038/s41467-019-10210-3

10. Jain C, Rodriguez-R LM, Phillippy AM *et al.* High throughput ani analysis of 90k prokaryotic genomes reveals clear species boundaries. *Nat Commun*. 2018;**9** https://doi.org/10.1038/s41467-018-07641-9

11. Hitch TCA, Riedel T, Oren A *et al.* Automated analysis of genomic sequences facilitates high-throughput and comprehensive description of bacteria. *ISME Commun*. 2021;**1**:16 https://doi.org/10.1038/s43705-021-00017-z
